# Items

**Published:** 1882-01

**Authors:** 


### Items.

GENERAL RULES FOR THE PREVENTION AND RESTRICTION OF CONTAGIOUS DISEASES. Issued by the Michigan State Board of Health.

1. Avoid the contagium or special cause of the disease. Do not take the breath of one sick. Unless you are needed to care for the sick, or are protected by having had the disease, or, in case of small-pox, by thorough vaccination, do not go near the sick person. Do not allow your lips to touch any food, cup, spoon, or anything else that the sick person has touched or that has been in the sick room. Do not wipe your face or hands with any cloth that has been near the sick person. Do not wear any clothing the sick person has worn during, just before, or just after his sickness. Keep your hands free from discharges from the body or skin of the sick person. Do not touch him with sore or scratched hands. Particularly avoid inhaling or in any way receiving into the mouth or nose the branny scales that fall off or peel off from one recovering from, or apparently wholly recovered from scarlet fever.

2. Restrict the contagium or special cause of the disease. Isolate the sick. Separate those sick with any of these diseases, even if they are but mildly sick, from all persons except necessary attendants. A person sick with any of these diseases should not be permitted to suffer from want of care, food, or comfort; but all his wants should be attended to by adults, or by those who are protected by proper vaccination or by having had the disease. Children, and those who are not thus protected, should be kept away from these diseases. Do not go from the sick-room to a child or other unprotected person until after change of clothing, and thorough washing of hands, face, hair and beard. Always wash the hands thoroughly after any necessary handling of the sick person, or of anything that has been in contact with the sick. Keep those who have been exposed to any of these diseases away from schools, churches and other assemblies, and from all children, until it is known whether they are infected—and if they are found to be infected, isolate them till after complete recovery and thorough disinfection.

3. Destroy the contagium or special cause of the disease,—

*a.* By thoroughly disinfecting or destroying whatever is removed from the person sick or from the sick-room. All discharges from the patient should be received into vessels containing a strong solution of sulphate of iron (copperas), and then, in cities, thrown into the water-closet; elsewhere they should be buried at least one hundred feet distant from any well; or where this is impracticable they should be received on old clothes, which should immediately be burned or disinfected and buried.

*b.* By thoroughly disinfecting the sick-room and its contents, after removal of the sick person, whether by death or recovery.

Disinfect as follows: Burn whatever has been in contact with the sick person and is not too valuable to burn. Garments, sheets, blankets, etc., that will not be injured by bleaching, should be boiled for half an hour in a zinc-solution made by dissolving zinc sulphate and common salt in water, in the proportion of four ounces of the zinc sulphate and two ounces of common salt to one gallon of water. Hang up and loosely spread out clothing, bedding, etc., that cannot be boiled in the zinc-solution, or spread it loosely over chairs in the sick-room, leaving the bedstead and other furniture in the room. Close all openings to the room very tight. For a room ten feet square place two pounds of sulphur in an iron pot or pan supported on bricks. Set the sulphur on fire with live coals or with a spoonful of alcohol lighted by a match. Be careful not to breathe the sulphurous fumes. Leave the room tightly closed for several hours, then air it thoroughly. For a large room use a proportionately larger quantity of sulphur at the rate of two pounds for each 1,000 cubic feet of air-space, and try to burn as much as possible of the sulphur used.

4. Keep your house and premises and everything connected, therewith clean, but remember that *the contagium of these diseases may attach to the cleanest article* of clothing, food, drink, book, or paper, if it is exposed thereto.

TREATMENT OF FELONS. — Adinell Hewson offers some suggestions for the accurate diagnosis and successful treatment of felons. For diagnosis he makes a flattened conical tube of binder's board with its base five by three and one half inches in diameter, so trimmed as to fit closely over brow, cheeks and upper lip. The length is such as to bring the apex at about the distance of the range of distinct vision. The apex is an orifice one-eighth by three-sixteenths of an inch in diameter. The tube is made from a sheet of binder's board by dipping in warm water to soften it, then rolling it diagonally, and wrapping with cord to retain form until dry.

By means of this simple apparatus he examines the tissues by transmitted light. In the case of a suspected felon the patient's finger is brought to the point of the tube, which is held in the direction of a bright light, either natural or artificial, while the face is so applied at the base as to make it fit closely and exclude the light. During the examination Dr. Hewson finds it of advantage to have the patient practice forced, rapid respiration to produce an anæsthetic effect. If the apex of the tube covers healthy tissues of the finger, the characteristic bright pinkish-red color is readily perceived, while if the tissues are engorged the darker red tint, deepened in proportion to the intensity of the engorgement, will be equally characteristic, and will form a marked contrast to the color to be seen on examining the corresponding finger on the other hand. If the tint, though still reddish, be of a yellow hue pus has formed in the cellular tissue around or in the theca of the tendon. If by making firmer pressure, so as to cut off the lateral illumination through the tissues, the tint is found to be of a positive yellow, it is evident that there is suppuration in the theca of the tendon. Finally, if the tint so transmitted is of a dirty or opaque yellow the bone or periosteum is the seat of purulent formation and collection.

When such examination demonstrates that pus has not yet formed, he has generally succeeding in aborting a felon by the application of a thick paste of wet clay, covered first with tissue paper and then with a thin layer of bandage stiffened by liquid glue painted in strips lengthwise on each side of the finger. The object of applying the glue thus, instead of covering the whole surface, is to allow the drying of the clay, which would be prevented by coating the whole surface with glue.

Dr. Hewson's experience with such uses of clay has been very extensive, and he reports some very interesting and valuable results obtained by this agent. In this class of cases here considered he finds that as a rule the relief is very prompt, in which case the dressing is allowed to remain for several days. When the pain is not relieved in two or three hours after the application of the earth he removes it at once and makes a free incision, as he feels sure that nothing else will arrest the process.—*Coll. and Clin. Record.*

A Blind Horse for a Surgeon.— Dr. Geo. I. Rice, of La Moille, Illinois, relates a very interesting case. A patient presented himself with an old dislocation of the shoulder downward, which had occurred nearly a year before. No force, which it was prudent to use, was sufficient to reduce it, and the case was left to nature.

Last Thanksgiving day, just four years after the joint was dislocated, the patient was riding in a buggy, leading a blind horse following. To make sure of his hold, he wound the halter around his wrist. The blind horse chancing to run against the hind wheel, became frightened and jerked violently backward. The forward horse meanwhile kept pulling ahead. The wrench was terrible, and the man went home nearly sick and took to bed. At length he fell asleep, and woke after some hours refreshed. To his great surprise, he found the dislocated shoulder reduced, and in a short time the joint regained nearly its normal usefulness.

It is believed that the Board of Health will have this horse fined for practicing without a license.

